# Comparative Proteomic Analysis of Different Isolates of *Fusarium oxysporum* f.sp. *lycopersici* to Exploit the Differentially Expressed Proteins Responsible for Virulence on Tomato Plants

**DOI:** 10.3389/fmicb.2018.00420

**Published:** 2018-03-06

**Authors:** Rajendran Manikandan, Sankarasubramanian Harish, Gandhi Karthikeyan, Thiruvengadam Raguchander

**Affiliations:** Department of Plant Pathology, Centre for Plant Protection Studies, Tamil Nadu Agricultural University, Coimbatore, India

**Keywords:** fungal proteomics, *Fusarium wilt*, 2D-page, MALDI-TOF, pathogenicity

## Abstract

The vascular wilt of tomato caused by *Fusarium oxysporum* f.sp. *lycopersici* is an important soil borne pathogen causes severe yield loss. The molecular characterization and their interaction with its host is necessary to develop a protection strategy. 20 isolates of *F. oxysporum* f.sp. *lycopersici* (FOL) were isolated from wilt infected tomato plants across Tamil Nadu. They were subjected to cultural, morphological, molecular and virulence studies. The results revealed that all the isolates produced both micro and macro conidia with different size, number of cells. The colors of the culture and growth pattern were also varied. In addition, chlamydospores were observed terminally and intercalary. The PCR analysis with *F. oxysporum* species-specific primer significantly amplified an amplicon of 600 bp fragment in all the isolates. Based on the above characters and pathogenicity, isolate FOL-8 was considered as virulent and FOL-20 was considered as least virulent. Proteomics strategy was adopted to determine the virulence factors between the isolates of FOL-8 and FOL-20. The 2D analyses have showed the differential expression of 17 different proteins. Among them, three proteins were down regulated and 14 proteins were significantly up regulated in FOL-8 than FOL-20 isolate. Among the 17 proteins, 10 distinct spots were analyzed by MALDI-TOF. The functions of the analyzed proteins, suggested that they were involved in pathogenicity, symptom expression and disease development, sporulation, growth, and higher penetration rate on tomato root tissue. Overall, these experiments proves the role of proteome in pathogenicity of *F. oxysporum* f.sp. *lycopersici* in tomato and unravels the mechanism behinds the virulence of the pathogen in causing wilt disease.

## Introduction

The vascular wilt incited by *Fusarium oxysporum* f.sp. *lycopersici* (FOL) is a devastating disease of tomato in major growing regions worldwide. Until now, three host specific races (races 1, 2, and 3) of this pathogen have been reported. Among the races, race 1 is the most widely distributed and has been reported from most geographical areas (Reis et al., 2004). The pathogen is a soil inhabitant. It survives in soil and in infected plant debris present between crop rotations as mycelium and it produce chlamydospores under cooler temperate regions. This pathogen infects the tomato plants through mycelium or penetrating tip of the roots by spore germination, wounds from roots or root laterals. The hyphae enter into the root cortex and xylem by means of intracellularly. The production of microconidia from the mycelium takes place exclusively in the xylem vessels. Movement of microconidia occurs upward by entering into the sap stream of roots. Finally, the mycelium along with spores clogs the vascular vessel and arrests the plant from absorption and nutrient translocation. Further, plant transpires more than it can transport, resulting in closure of stomata, wilting and cause plant death. The fungus penetrates all tissues after death of the plants and sporulates for continues infection of adjoining plants. Production of fusaric acid by this pathogen considered as a potent pathogenicity factor for the development wilt disease in tomato (Singh et al., 2017).

Traditionally, FOL are differentiated by their morphological characteristics on selective media but not in species level variations (Booth, 1975). The virulence profile of the fungus can be determined based on the ability to infect different tomato cultivars possessing multiple resistance genes (Tanyolaç and Akkale, 2010). Efficient and proper disease management strategies required better understanding of molecular biology of the fungus, pathogenicity and host-pathogen interactions. It can be achieved by using different techniques, one of them is proteomics. Now a days it become a potential tool for obtaining molecular information about pathogenicity and factors involved in virulence, thus opening up new insights for plant disease detection and protection. This technique allows the measurements of large numbers of proteins both qualitative and quantitatively that directly influences cellular biochemistry and it provides exact analysis of cellular state or structural changes during growth, development under different environmental conditions. In the recent years, field of fungal proteome is gathering pace as a result of the simultaneous occurrence of full length genome sequence of the fungus availability and advances in high sensitivity protein mass spectrometry.

In phytopathogenic fungi, proteomic approaches was very useful tool for identification of differentially expressed proteins in fungal species (Wang et al., 2011). First effector protein with molecular weight of 12 kDa cysteine-rich protein was identified as root invader in tomato *F. oxysporum* pathosystem (Rep et al., 2004). An analysis of protein profile of two *Botrytis cinerea* strains showed the difference in virulence as well as toxin production level in both qualitative and quantitatively. Among the differential protein expressions, few of the proteins were dehydrogenase and glyceraldehyde-3-phosphate dehydrogenase and a cyclophilin, proteins and they were involved in virulence (Fernández-Acero et al., 2006). Mycelial proteomes of *Sclerotinia sclerotiorum* (Yajima and Kav, 2006) were described using 2-DE reference maps for identification of proteins. Proteomic strategy were adopted in corn smut fungus caused by *Ustilago maydis* and it dissects the proteins involved in the transition from budding to filamentous growth, which makes the fungus in to a pathogenic form (Böhmer et al., 2007).

Proteomics can also been adopted for successful analysis of metabolic pathways such as synthesis of mycotoxin from *Fusarium graminearum* (Taylor et al., 2008) and cellulose degradation by *B. cinerea* (Fernández-Acero et al., 2010). Secreted proteins are often potential interest in phytopathogenic fungi due to their importance in host-pathogen interactions. Analyses of proteins secreted from *F. graminearum* upon growth in different media and infection time of wheat heads showed that the proteins played an important role in host pathogen interaction (Paper et al., 2007). Comparative proteome analysis in fungal species and its strains has contributed to the understanding of the infection process, wide host range, pathogenicity, metabolisms, stress response, signal transduction and identification of candidate virulence proteins (Xu et al., 2007). El-Bebany et al. (2010) identified fungal proteins that played roles in stress response, mycelia colonization, biosynthesis of melanin, sclerotic formation, resistance against antibiotics and penetration in the plants. Olja et al. (2012) identified 10 proteins from brown rot fungus caused by *Monilinia laxa* in apple and concluded that they are potential host specific candidates for the development of *M. laxa*. Miguel-Rojas and Hera (2013) exploited the proteomic approach in *F. oxysporum* f.sp. *lycopersici* and identified the differential pattern of proteins were involved in vesicle blocking and proteasome degradation. Proteins from different functional classes are severely affect the tomato leaf tissues upon fusaric acid exposure resulted in deterioration of structure plant cell metabolism (Singh et al., 2017).

With this background information, the present study was carried out to characterize and determine the virulence among the isolates of *F. oxysporum* f.sp. *lyocpercisi* that cause wilt disease in tomato using proteomic approach.

## Materials and methods

### Isolation and pathogen identification

The tomato plants showing typical wilt symptoms were collected from different farmer's field in different districts of Tamil Nadu (Table 1). The pathogen was isolated from infected root portions. The pure culture of the pathogen was obtained by single hyphal tip method (Rangaswami, 2005) and 20 different fungal isolates were named with the codes of FOL 1–20.

**Table 1:** List of *Fusarium oxysporum* f.sp. *lycopersici* isolates from different places of Tamil Nadu.

**S No**.	**District**	**Location**	**Isolate code**
1.	Dindugal	Nilakottai	FOL-1
2.	Dindugal	Ottanchatram	FOL-2
3.	Karur	Parmathi	FOL-3
4.	Karur	Mailampatti	FOL-4
5.	Tirupur	Moolanur	FOL-5
6.	Tirupur	Udumalapet	FOL-6
7.	Coimbatore	Kinathukadavu	FOL-7
8.	Coimbatore	Thondamuthur	FOL-8
9.	Erode	Vellakovil	FOL-9
10.	Erode	Bhavani	FOL-10
11.	Namakkal	Rasipuram	FOL-11
12.	Namakkal	Tiruchengodu	FOL-12
13.	Salem	Aattayampatti	FOL-13
14.	Salem	Tarmangalam	FOL-14
15.	Dharmapuri	Kasiampatti	FOL-15
16.	Dharmapuri	Pudur	FOL-16
17.	Krishnagiri	Maharajakadai	FOL-17
18.	Krishnagiri	Kochampalli	FOL-18
19.	Vellore	Ambur	FOL-19
20.	Vellore	Pudupadi	FOL-20

### Phenotypic characterization of *F. oxysporum* f.sp. *lycopersici*

Totally 20 *F. oxysporum* f.sp. *lycopersici* isolates were grown on Potato dextrose agar (PDA) medium to study their growth and variability in colony characters. From the 8 day old culture plates, 5 mm disc of the fungus was placed at the center of each sterile Petri dish containing 15 ml of sterilized and solidified PDA. The cultures were incubated at room temperature (28 ± 2°C) for 7 days. The mycelial growth, colony characters, number of spores and its characters were recorded 7 days after inoculation (DAI).

### Determination of virulence and pathogenicity of *F. oxysporum* f.sp. *lycopersici* on tomato plants

All the isolates of *Fusarium* wilt pathogen was multiplied in sand maize medium. The four fungal discs was uniformly inoculated into sand maize medium and incubated for 15 days at room temperature (28 ± 2°C) for better multiplication (Riker and Riker, 1936). Potting soil (Red soil: sand: cow dung manure @ 1:1:1 w/w/w) was sterilized. Virulence and pathogenecity of the 20 isolates was tested by artificially inoculating the sand maize culture in the potting soil at 10 per cent w/w which ensured the spore concentration of 6.5 × 10^5^. The potting soil incorporated with the fungus was filled in 30 cm diameter pots. Seeds of tomato cv. Co-3 were surface sterilized with 0.1 per cent mercuric chloride solution for 30 s and rinsed three times with sterile distilled water and sown in the pro-tray. Totally three plants per pot with three replications were adopted for this study. After 25 days the seedlings were transplanted in to 30 cm diameter pots and irrigated properly with glass house water systems. The observation on disease incidence was monitored regularly for symptom expression, virulence and pathogenecity. The disease incidence was assessed using the following formula

$$\begin{array}{l} \text{Per cent disease Incidence}=\frac{\text{Number of infected plants}}{\text{Total number of plants}}\;\times \;100 \end{array}$$

For determination of virulence, disease severity was estimated using an index of leaf damage (ILD) described by Beye and Lafay (1985). The formula of the scale was 0–4 (0 = asymptomatic leaves, 1 = leaves wilted, 2 = leaves with moderate yellowing, 3 = leaves with necrosis, and 4 = dead leaves). The ILD was calculated according to the following formula, ILD = grades/max grade. Based on the ILD scores, tested isolates were categorized as less virulent (+), moderately virulent (++), and highly virulent (+ + +).

### Molecular characterization of *F. oxysporum* f.sp. *lycopersici* isolates DNA isolation and PCR amplification

The 10 days old cultures of each *F. oxysporum* f.sp. *lycopersici* were transferred into 250 ml conical flasks containing 150 ml potato dextrose broth (PDB) and incubated at room temperature for 7 days. Mycelium was harvested by filtration through sterile filter and used for DNA extraction using standard CTAB method.

**Figure 1:**
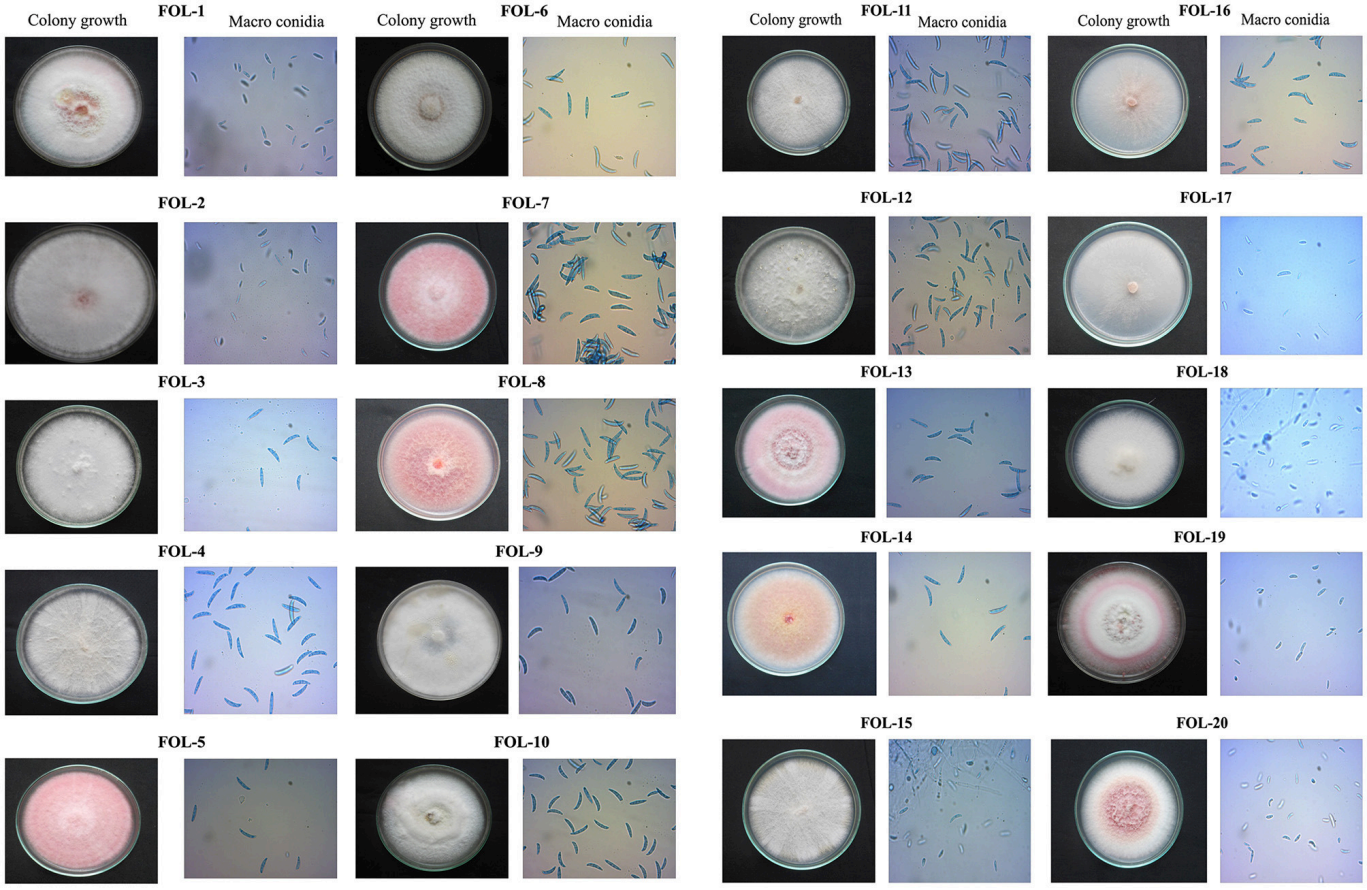
Cultural and morphological characters of *F. oxysporum* f.sp. *lycopersici*.

**Figure 2:**
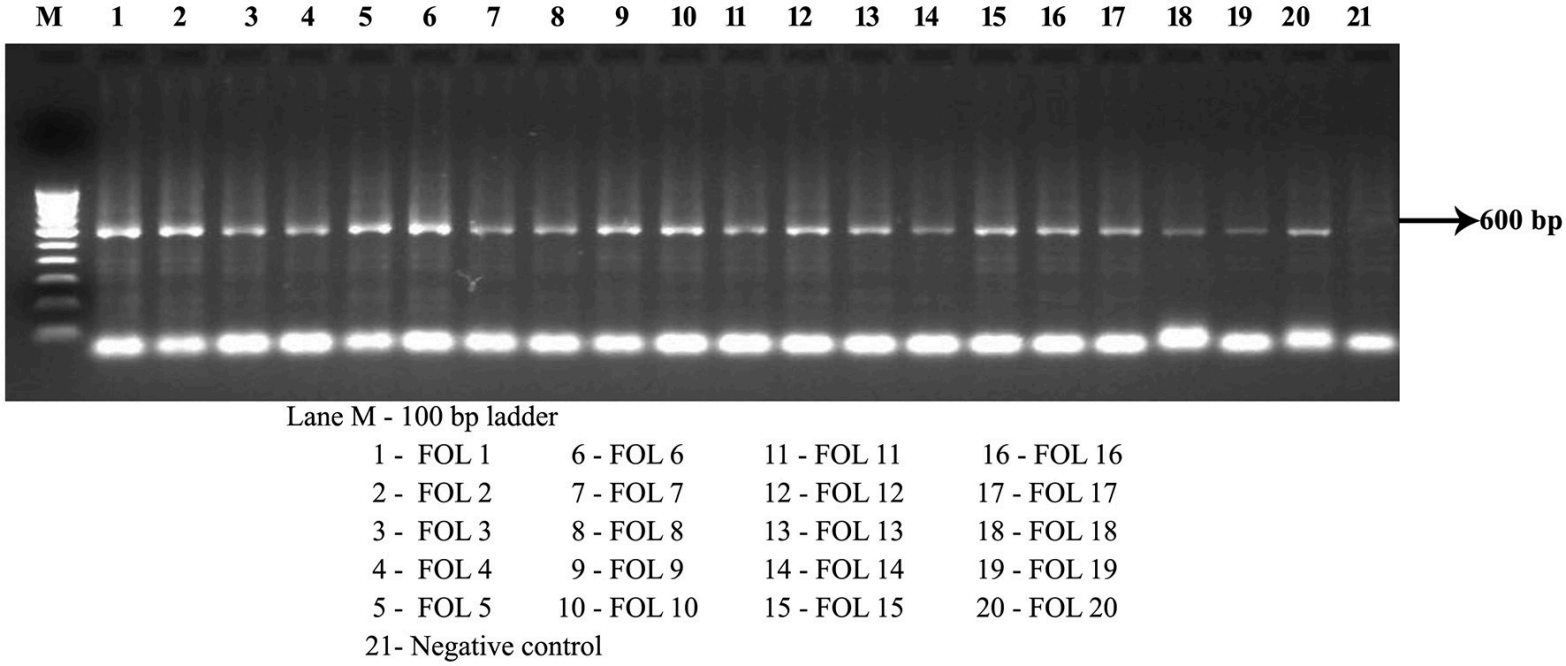
PCR amplification of intergeneric region of *F. oxysporum* f.sp. *lycopersici* using species specific primers IGS 1 and IGS 2.

**Figure 3:**
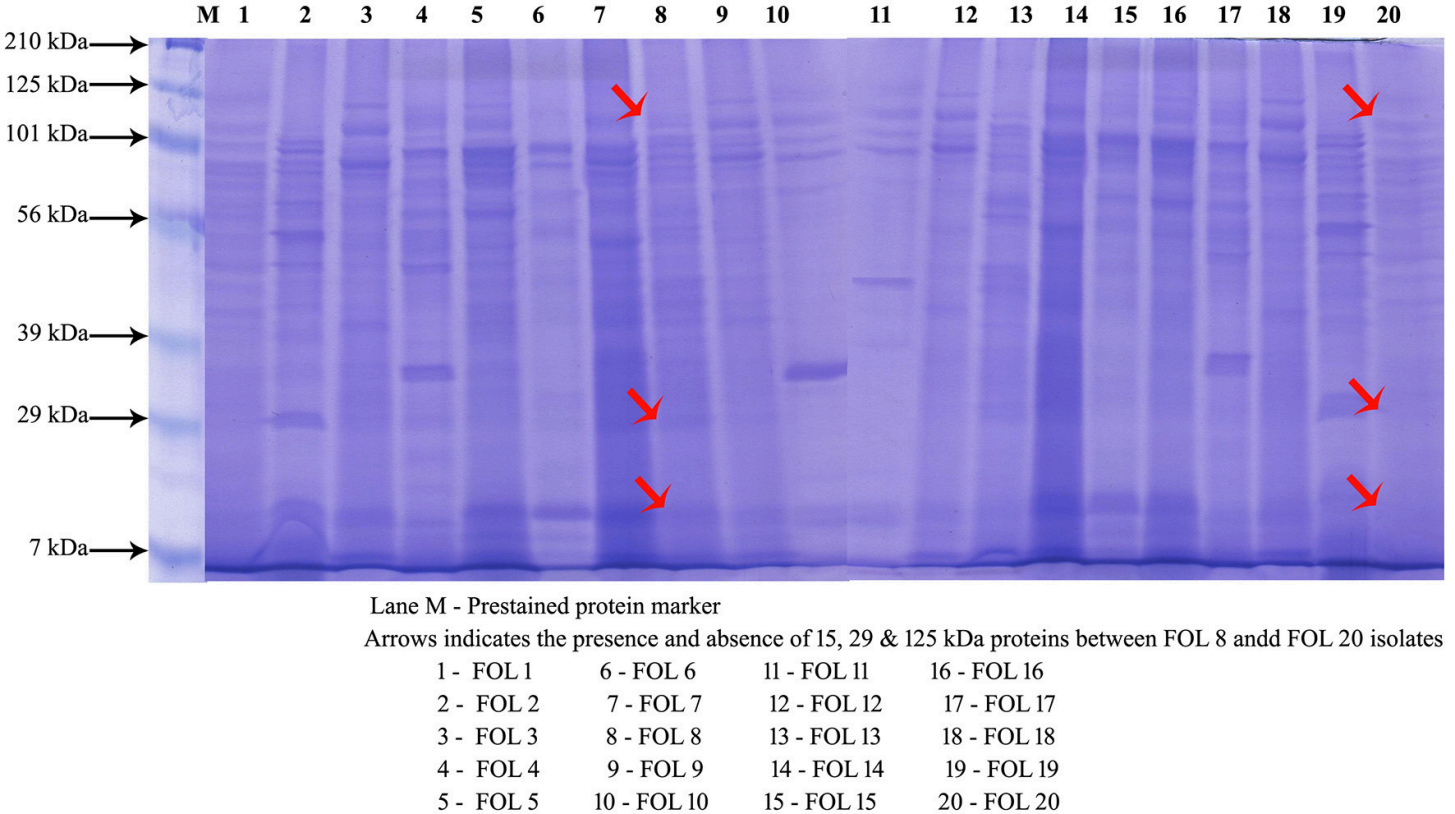
Protein profiling of *F. oxysporum* f.sp. *lycopersici* isolates by SDS-PAGE.

**Figure 4:**
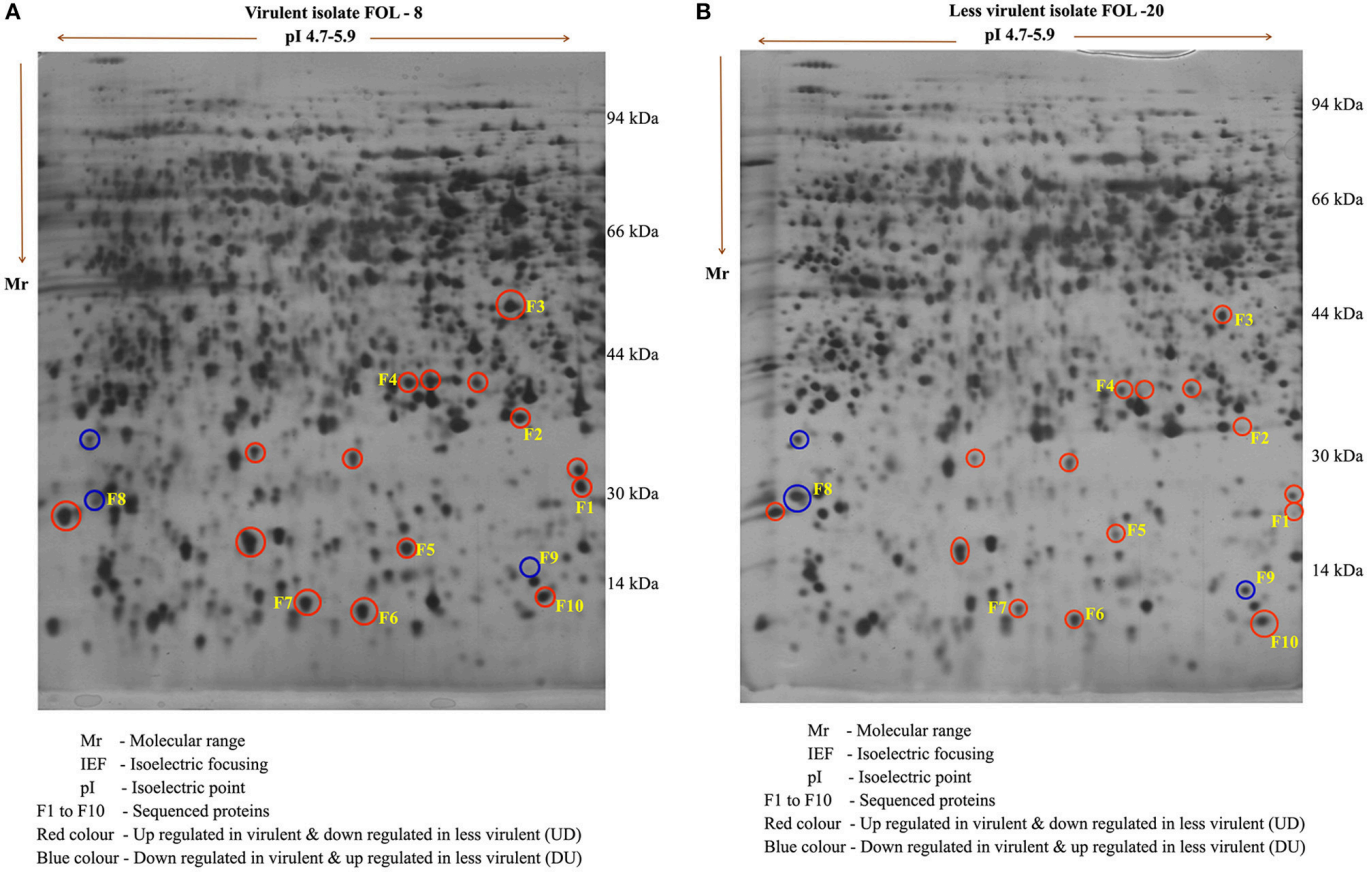
Protein profile of *F. oxysporum* f.sp. *lycopersici* in virulent (FOL-8) and less virulent (FOL-20) isolates by 2D – gel electrophoresis. **(A)** Virulent isolate FOL – 8. **(B)** Less virulent isolate FOL – 20.

**Figure 5:**
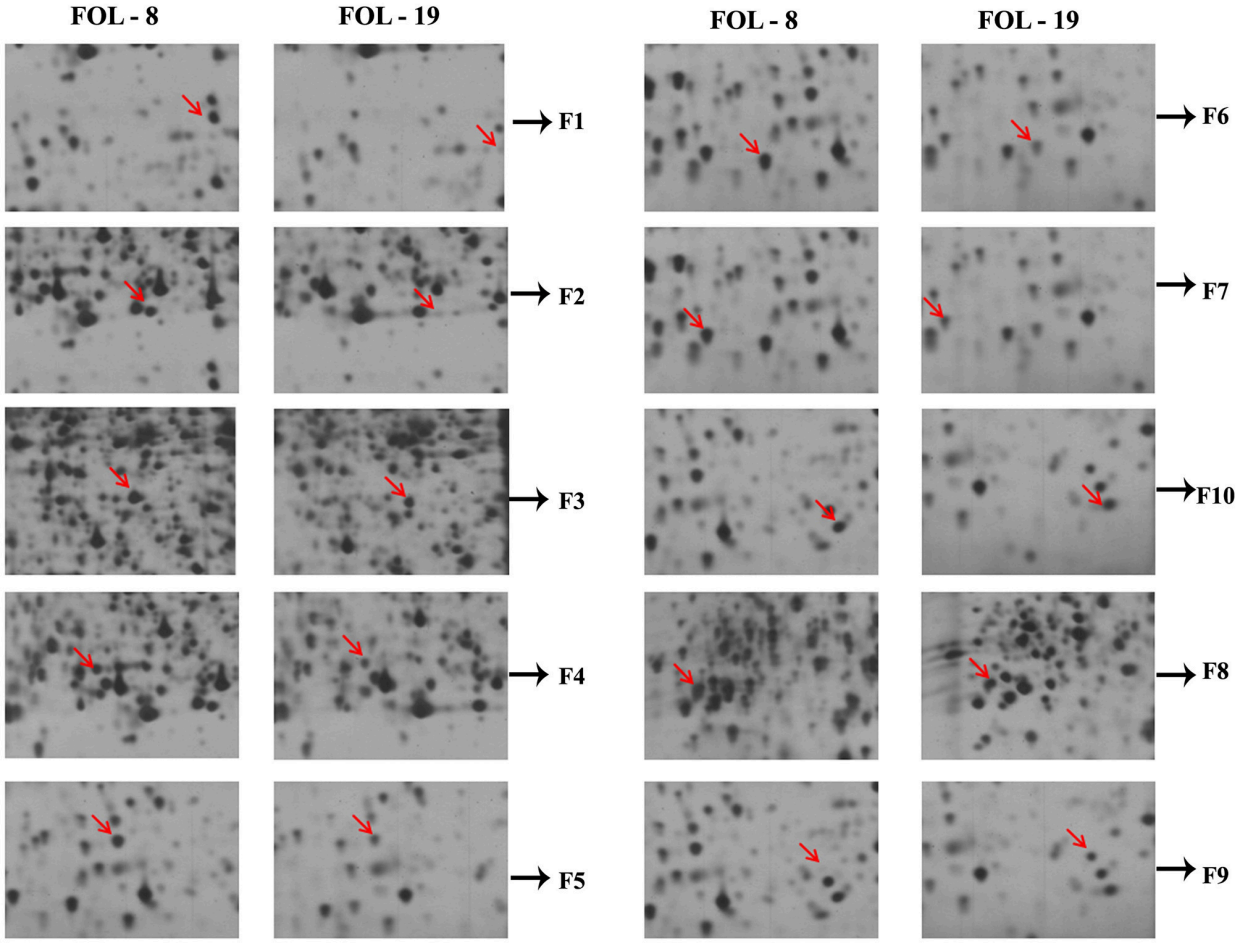
Differential expression of virulent proteins in *Fusarium oxysporum* f.sp. *lycopersici* isolates causing wilt disease in tomato.

PCR assay was carried out using total DNA from FOL isolates as templates to amplify the ribosomal DNA intergenic spacer (IGS) region in the FOL genome. The primers FIGS 1 - 5′- GTAAGCCGTCCTTCGCCTCG-3′ and FIGS 2 - 5′- GCCATACTATTGAAT TTT GC-3′ were used to amplify ribosomal DNA intergenic spacer regions of *F*. *oxysporum* f.sp. *lycopersici* of 20 isolates along the positive control obtained from Department of Plant Pathology, Tamil Nadu Agricultural University, Coimbatore, India. PCR reaction was performed with intial denaturation step at 94°C for 5 min, 35 cycles of amplification (30 s for denaturation at 94°C, 30 s for primer annealing at 59°C and 1 min for extension at 72°C) and one cycle of final extension at 72°C for 7 min in PCR Thermo Cycler. The amplified PCR products were run on 1.5% agarose gel and visualized on an UV-transilluminator and photographed in the gel documentation unit.

### Protein profiling of FOL isolates by SDS-page

One ml of 0.1M sodium phosphate buffer (pH 7.0) was used for extraction of proteins from 1 g of powdered mycelia under 4°C. The supernatant was collected from the homogenate by centrifugation for 20 min at 10,000 rpm and used for the SDS-PAGE (Laemmli, 1970). One hundred micrograms of protein from each isolates was taken and mixed with 10 μl of sample buffer in a microfuge tube, boiled for 4 min and incubated at 4°C for 30 min. Then the samples containing equal amount of proteins were loaded into the wells of polyacrylamide gels. The medium range protein markers were used for electrophoresis and gels were stained with 0.2% Coomassie brilliant blue (R250) solution.

### 2D-page analysis in virulent (FOL-8) and less virulent (FOL-20) isolates

Two dimensional electrophoresis was carried out to determine the virulence factors between virulent isolate (FOL-8) and less virulent isolate (FOL-20). Three biological replicates of each isolate were carried out in the experiment. The 10 days old cultures of FOL-8 and FOL-20 were grown in PDB and incubated at room temperature for 7 days. Mycelium was harvested by filtration through sterile filter and used for protein extraction. Mycelium was grounded using liquid nitrogen and immersed in 10% trichloracetic acid (TCA) in acetone with 0.07% dithiothreitol (DTT) at −20°C for 1 h, followed by centrifugation for 20 min at 12,000 rpm. The pellets were washed once with ice cold acetone containing 0.07% DTT at −20°C for 1 h and centrifuged again for 20 min at 12,000 rpm. Clear supernatant was obtained by repeating three times of washing step.

The final pellet powder were placed in a −80°C freezer until frozen, 1 h and then placed in a lyophilizer until the pellet becomes a dry powder. A total of 10 mg of the dried powder was dissolved in 250 μL of sample buffer containing 7M urea, 2M thiourea, 4% 3-[(3-cholamidopropyl) dimethylammonio]-1-propanesulfonate (CHAPS), 0.5% ampholytes (Bio-rad) and 0.7% DTT. The pellets was extracted using extraction buffer was gently shaken for 1 h then centrifuged for 20 min at 12,000 rpm at room temperature. The supernatant was distributed in 100 μL aliquots and kept at −80°C before 2D-PAGE analysis.

For analytical gels, the 17 cm IPG (Immobiline pH Gradient) strips (4.7–5.9 pH) were rehydrated for 12 h with 350 μl of rehydration buffer (8M Urea, 2% CHAPS, DTT (7 mg per 2.5 ml of rehydration buffer) and 0.5% (v/v) IPG buffer pH 4–7) containing the sufficient quantity of proteins (150 μg) in a reswelling tray at room temperature. The strips were allowed for about 12–14 h for rehydration and subjected to the first dimension separation. Isoelectric focusing (IEF) was carried out at 20°C with a Biorad IEF cell. The running conditions were as follows: 500 V for 1 h followed by 1,000 V for 1 h and finally 3,000 V for 12 h. The focused strips were equilibrated twice for 15 min in 10 ml equilibration solution. The first equilibration was performed in a solution containing 6M urea, 30% (w/v) glycerol, 2% (w/v) SDS, 1% (w/v) DTT and 50 mM Tris-HCl buffer. The second equilibration was performed in a solution modified by the replacement of DTT by 4% (w/v) iodoacetamide.

Separating gel (30%) was poured between the glass plates. Equilibrated IPG strips were rinsed with electrode buffer and placed on the top of SDS gel and overlaid with 2 ml of agarose solution. Gels were electrophoresed at constant current (15 mA) till the dye front reaches the bottom of the gel. Gels were removed from their gel cassettes and fixed in the fixative (4:1:5 of methanol:acetic acid:water). The gels were stained by silver staining method as described by Blum et al. (2005). After fixing for 1 h, gels were washed (30% Ethanol) twice for 20 min each. Third washing was done with deionized water. Gels were sensitized for 1 min in 0.02% thiosulfate reagent. Gels were washed three times in deionized water (30 sec each) and impregnated in 0.2% Silver nitrate solution for 30–60 min. The gels were developed after thorough washing using deionized water (three times) using a developer (30 g sodium carbonate and 5 mg of sodium thiosulfate in 1,000 ml water) for 8–10 min. The reaction was stopped using a stopper reagent and rinsed again with deionized water.

Silver stained gels were scanned using Chemicdoc XRS system (Bio-rad). Selected spots were picked from the gels (Salekdeh et al., 2002). The data from MALDI-TOF MS were searched against the fungi databases with MASCOT and Profound softwares. The following parameters were used for database searches: Taxonomy: Fungi, cleavage specificity, trypsin with 1 missed cleavages allowed, Peptide tolerance of 100 ppm – 300 ppm for the fragment ions; allowed modifications, Cys Carbamidomethyl (fixed), oxidation of Met (variable). The peptide mass fingerprinting of the proteins were scored with the Mowse score.

### Statistical analysis

The data were statistically analyzed using the IRRISTAT version 92 developed by the International Rice Research Institute Biometrics Unit, the Philippines (Gomez and Gomez, 1984). Prior to statistical analysis of variance (ANOVA) the percentage values of the disease incidence were arcsine transformed. Data were subjected to analysis of variance (ANOVA) at two significant levels (*P* < 0.05 and *P* < 0.01) and means were compared by Duncan's Multiple Range Test (DMRT).

## Results

### Phenotypic characterization of *F. oxysporum* f.sp. *lycopersici* isolates

Microscopic examination revealed that all the isolates produced two types of conidia namely micro and macro conidia. Micro conidia are small, oval shaped, hyaline, and single or bicelled. In case of macroconidia were sickle shaped hyaline and multicelled with three to five septate. The number of microconidia was more as compared to macro conidia. In addition chlamydospores were observed terminally and intercalary.

Based on sporulation, the isolates were divided into three category *viz*., poor sporulation, medium sporulation and good sporulation. Twelve isolates *viz.*, FOL-7, FOL-8, FOL-9, FOL-10, FOL-11, FOL-12, FOL-13, FOL-14, FOL-15, FOL-16, FOL17, and FOL-18 had produced good sporulation while four isolates *viz.*, FOL-3, FOL-4, FOL-5, and FOL-6 produced sporulation at medium level. Least sporulation and growth was recorded in four isolates namely FOL-1, FOL-2, FOL-19, and FOL-20. Colony characters of each isolates showed that they were significantly different from each other. At the time of incubation pathogen produced different colony colors *viz*., light pink, pink, dark pink, creamy white, pale white with pink, creamy white with pink, milky white, and pinkish white. The result of mycelial growth pattern showed two different pattern namely adherent smooth (9 isolates) and fluffy growths (11 isolates) (Figure 1, Table 2).

**Table 2:** Cultural and morphological characters of different isolates of *Fusarium oxysporum* f.sp. *lycopersici*.

**Isolate No**	**Colony color**	**Mycelial Growth pattern**	**Sporulation**	**Growth type**	Length (μm)^*^	Width (μm)^*^
FOL-1	Light pink	Adherent smooth	+	Slow	26.5	3.4
FOL-2	Light pink	Adherent smooth	+	Slow	25.0	3.5
FOL-3	Milky white	Fluffy growth	++	Medium	34.5	3.8
FOL-4	Milky white	Fluffy growth	++	Medium	31.0	3.5
FOL-5	Pink	Fluffy growth	++	Medium	29.0	3.7
FOL-6	Light pink	Fluffy growth	++	Medium	31.0	3.7
FOL-7	Light Pink	Fluffy growth	+++	Fast	36.0	3.4
FOL-8	Dark Pink	Fluffy growth	+++	Fast	38.7	4.0
FOL-9	Milky white	Adherent smooth	+++	Fast	32.5	4.1
FOL-10	Creamy white	Adherent smooth	+++	Fast	30.0	3.9
FOL-11	Creamy white	Adherent smooth	+++	Fast	32.1	4.2
FOL-12	Creamy white	Adherent smooth	+++	Fast	31.1	4.3
FOL-13	Creamy white with pink	Fluffy growth, smooth	+++	Fast	26.7	3.6
FOL-14	Creamy white with pink	Adherent growth	+++	Fast	29.0	3.7
FOL-15	Pale white with light yellow	Fluffy growth, smooth	+++	Fast	33.5	3.8
FOL-16	Pale white with light yellow	Fluffy growth	+++	Fast	31.0	3.7
FOL-17	Pure white	Fluffy growth	+++	Fast	32.5	3.9
FOL-18	Pure white	Fluffy growth	+++	Fast	32.0	3.9
FOL-19	Pinkish white	Adherent smooth	+	Slow	29.5	3.6
FOL-20	Pinkish white	Adherent smooth	+	Slow	28.0	3.5

+, Poor sporulation; 1–10 spores/microscopic field (100X); ++, Medium sporulation; 11–50 spores/ microscopic field (100X), +++ Good sporulation; More than 100 spores/ microscopic field (100X),

* *Mean of 10 conidia*.

### Pathogenicity, symptomatology, and virulence

Pathogenicity study for the soil borne pathogen, *F. oxysporum* f.sp. *lycopersici* isolates showed that the symptoms were expressed after 7 days of inoculation in tomato variety tomato cv. Co-3. The symptoms were characterized as the first appearance of slight vein clearing on the younger leaves of outer portion and further showing the epinasty in the older leaves and stunting of the plants, lower leaves shows yellowing, adventious roots formation, leaves and young stems wilting, defoliation, marginal necrosis of rest of the leaves and finally death of the entire plant.

All the isolates produced significant symptoms under pot culture studies and percent disease incidence (PDI) ranged from 52.50 to 100 was noticed. The isolate FOL-8 recorded highest PDI (100%) followed by FOL-9. The least incidence of PDI (48.00) was recorded in FOL-20 isolated followed by FOL-19 (52.50). From these results, it was clear that the isolates FOL-8 and FOL-9 were considered to be virulent and FOL-19 and FOL-20 considered being less virulent (Table 3).

**Table 3:** Determination of virulence based on the pathogenicity of *F. oxysporum* f.sp. *lycopersici* isolates under glasshouse conditions.

**S. No**.	**Isolate code**	**Symptom expression at DAT**	**Virulence type**	**Wilt incidence %**
1.	FOL-1	80	+	51.00^i^ (45.57)
2.	FOL-2	80	+	54.00^hi^ (47.29)
3.	FOL-3	60	++	75.20^de^ (60.17)
4.	FOL-4	58	++	70.00^ef^ (56.81)
5.	FOL-5	65	+	55.00^ghi^ (47.87)
6.	FOL-6	65	+	51.15^i^ (45.65)
7.	FOL-7	46	+++	95.00^b^ (78.67)
8.	FOL-8	45	+++	100.00^a^ (86.17)
9.	FOL-9	55	++	48.00^i^ (43.85)
10.	FOL-10	54	++	52.00^hi^ (46.14)
11.	FOL-11	54	++	60.00^gh^ (50.77)
12.	FOL-12	55	++	63.00^fg^ (52.54)
13.	FOL-13	52	++	52.00^hi^ (46.14)
14.	FOL-14	50	++	54.00^hi^ (47.29)
15.	FOL-15	51	++	50.00^i^ (49.99)
16.	FOL-16	51	++	55.50^ghi^ (48.16)
17	FOL-17	48	+++	90.00^c^ (71.87)
18.	FOL-18	47	+++	80.00^d^ (63.50)
19.	FOL-19	85	+	52.50^hi^ (46.43)
20.	FOL-20	85	+	48.00^i^ (43.85)

+, Less virulent; ++, Moderately virulent; +++, Highly virulent; DAT: days after transplanting. Means followed by a common letter are not significantly different at 1% level by DMRT ^*^Values in parentheses are arscine transformed values.

### PCR amplification

*F. oxysporum* species-specific primer amplified a fragment of 600 bp in all the isolates including positive control. However no amplification was obtained in negative control (without template DNA). From these result all the 20 isolates were tentatively identified as *F. oxysporum* f.sp. *lycopersici* (Figure 2).

### Protein profile of FOL isolates

The banding patterns of protein were predominant with more numbers in the isolate FOL-8 and which was significantly differing from other isolates. Similar banding pattern was observed in FOL-7 with less intensity of the proteins. Few numbers of proteins with less intensity was expressed in less virulent isolates FOL-19 and FOL-20. The proteins of 7, 29, 56, and 101 kDa were expressed in all the isolates. However, 15, 39, and 125 kDa proteins appeared in virulent isolates and absence of these proteins were observed in less virulent isolates (Figure 3).

### 2D-page analysis

The comparative analysis of proteins expressed in *F*. *o*. f.sp. *lycopersici* virulent (FOL-8) and less virulent (FOL-20) isolates revealed that there was significant variation among isolates in protein pattern. Approximately 300–350 clear and consistent protein spots were expressed in both the isolates. Among them, 17 spots were differentially expressed and they were categorized into two classes *viz*., up regulated in virulent isolate and down regulated in less virulent isolate (UD) and down regulated in virulent and up regulated in less virulent (DU) based on their differential regulation in the 2-D gels.

Out of 17, 10 well-focused spots were sequenced (F1-F10). Among 10 proteins, two proteins *viz*., F8 and F9 with the molecular weight of 22 and 28 kDa were found to be significantly down regulated in virulent isolate (FOL-8). Eight proteins in the pI range of 4.8–5.4 were found to be upregulated in virulent isolate (FOL-8), down regulated in less virulent isolate (FOL-20). In 10 proteins, F1 and F9 protein predominatly up regulated in virulent isolate whereas these proteins absent in less virulent isolate. Remaining spots F2, F3, F4, F5, F6, F7, F8, and F10 were present in both the isolates and these proteins were expressed at higher levels in virulent isolate of FOL-8 (Figures 4A,B).

**Figure 6:**
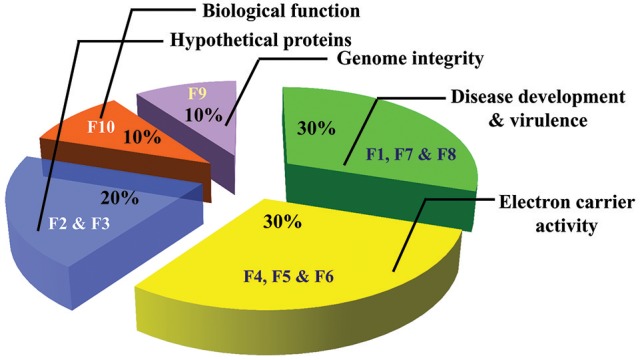
Pie charts of proteins identified from virulent and less virulent isolates and its functions (F1–F10 proteins spot codes).

A total of 10 proteins from 2-D acrylamide gels were excised and identified by MALDI-TOF mass spectrometry combined with database search using Mascot software. The functions of identified proteins were assigned by comparing their sequences to public protein database NCBInr. The identified proteins were Chaperone (gi|389744937), FAD binding domain containing protein (gi|347838696), Sulfate anion transporter (gi|471900325), Cytochrome P450 (gi|407916132), Cutinase-2 (gi|302408527), Glycoside hydrolase family 85 protein (gi|451995113), ATP-dependent RNA helicase (gi|402072144), 60S ribosomal protein (gi|615462440), and two were hypothetical proteins (gi|587722065 and gi|591452081). All identified proteins were identified with NCBI accession number of homology protein, score, experimental and theoretical pI-value and molecular weight (Table 4). All the identified proteins have broad spectrum activity, that is consistent with successful disease development, host-pathogen compatibility, electron carrier activity, catalytic activity, transfer of sulfate ions, hydroxylation and oxidation, breakdown of toxins, plant penetration and maintenance of genome integrity (Figures 5, 6, Table 5).

**Table 4:** List of expressed differential proteins in *Fusarium* isolates identified through 2-DE- MALDI-TOF.

**S. No**	**Spot ID**	**Coverage (%)**	**Score**	**Peptides Matched**	**Theoretical**		**Accession No**.	**Identity**	**Homologous protein**
					**pI**	**MW (kDa)**			
1.	F1	62	46	5	5.77	10.14	gi|389744937	Chaperone (*Stereum hirsutum*)	–
2	F2	72	44	4	5.78	84.82	gi|591404772	Hypothetical protein FOCG_17508 (*Fusarium oxysporum* f.sp. *radicis*-*lycopersici*)	–
3	F3	46	48	5	10.13	14.21	gi|591452081	Hypothetical protein FOPG_03417 (*Fusarium oxysporum* f.sp. *conglutinans* race 2)	–
4	F4	31	50	10	6.09	71.19	gi|347838696	FAD binding domain-containing protein (*Botryotinia fuckeliana*)	–
5	F5	87	50	4	4.74	86.74	gi|471900325	Hypothetical protein BN14_04229 (*Rhizoctonia solani*)	sulfate anion transporter (*Rhizoctonia solani*)
6	F6	51	46	4	5.9	15.5	gi|407916132	Cytochrome P450 (*Macrophomina phaseolina*)	–
7	F7	45	45	5	10.02	23.24	gi|302408527	Cutinase-2 (*Verticillium alfalfae)*	–
8	F8	28	62	13	5.43	78.30	gi|451995113	Glycoside hydrolase family 85 protein (*Bipolaris maydis*)	–
9	F9	66	67	7	6.46	25.68	gi|402072144	Hypothetical protein GGTG_14349 (*Gaeumannomyces graminis* var. *tritici*)	ATP-dependent RNA helicase SUB2 (*Magnaporthe oryzae*)
10	F10	55	45	4	8.09	76.37	gi|390600430	DUF1748-domain-containing protein (*Punctularia strigosozonata*)	–

**Table 5:** Functions of differentially expressed proteins from virulent (FOL-8) and less virulent (FOL-20) isolates of *F. oxysporum* f.sp. *lycopersici*.

**S. No**.	**Spot ID**	**Accession No**.	**Identified protein**	**Functions**
1.	F1	gi|389744937	Chaperone (*Stereum hirsutum*)	Requisite for successful disease development and for determining host-pathogen compatibility
2.	F2	gi|587722065	Hypothetical protein FOWG_06166 (*F. oxysporum* f.sp. *lycopersici*)	Unknown
3.	F3	gi|591452081	Hypothetical protein FOPG_03417 (*F. oxysporum* f.sp. *conglutinans*)	Unknown
4.	F4	gi|347838696	FAD binding domain containing protein (*Botryotinia fuckeliana*)	Electron carrier activity, oxidoreductase activity, and catalytic activity
5.	F5	gi|471900325	Sulfate anion transporter (*Rhizoctonia solani*)	Catalysis of the transfer of sulfate ions, SO4 from one side of a membrane to the other
6.	F6	gi|407916132	Cytochrome P450 (*M. phaseolina*)	Hydroxylation and oxidation processes and breakdown of toxins
7.	F7	gi|302408527	Cutinase-2 (*Verticillium alfalfae)*	Plant penetration and pathogenicity
8.	F8	gi|451995113	Glycoside hydrolase family 85 protein (*Bipolaris maydis*)	Detoxification, hydrolysis, or secondary metabolites biosynthesis
9.	F9	gi|402072144	ATP-dependent RNA helicase SUB2 (*Magnaporthe oryzae*)	Involved in transcription elongation and maintenance of genome integrity
10.	F10	gi|615462440	60S ribosomal protein L43 (*Colletotrichum fioriniae* PJ7)	Structural integrity and biological function of ribosomes

## Discussion

In the present study, wilt pathogen was isolated from infected tomato plants collected at different places of Tamil Nadu. All the isolates produced chlamydospores and two types of conidia namely micro and macro conidia was observed. Micro conidia are small, oval shaped hyaline and single or two-celled. Whereas macroconidia were sickle shaped, hyaline and multicelled with three to five septate. Similar results was observed by Rowe et al. (1977) who reported that, this fungus have three to five celled macrocodia with gradually pointed or curved edges on sporodochia in the diseased plant surface. Chlamydospores were usually developed as singly or in pairs, but it can also be found in clusters or short chains in some times. They were round and thick walled spores produced within or terminally in macroconidia or on older mycelium. The chlamydospores could survive in the soil for a long period of time (Rekah et al., 2000). The length and breadth of the macro conidia usually varied between 15–37.5 μm and 2.5–4 μm respectively in *F. oxysporum* f.sp. *lycopersici* isolates with 3 to 5 septation which was reported by Sonakar et al. (2013).

In the current study, Koch's postulates were proved for the wilt pathogen on tomato plants. The isolates of *F. oxysporum* f.sp. *lycopersici* were inoculated in sand maize media and was used as inoculum source. *F. oxysporum* f.sp. *lycopersici* was reisolated from artificially infected tomato plants (Manikandan et al., 2010). Ramamoorthy and Samiyappan (2001) reported that *F. oxysporum* f.sp. *lycopersici* in tomato produced the same symptoms during investigation. Browning of the vascular tissue is one of the characteristic symptoms of *Fusarium* wilt. Next, on aged plants, symptoms generally become more apparent in the period between blossoming and fruit maturation (Smith et al., 1988).

In our study, *F. oxysporum* f.sp. *lycopersici* isolates produced significant symptoms from 47 days after transplanting. The percent wilt incidence ranged from 48 to 100 between the isolates. This type of study was supported by Houssien et al. (2010) who noticed 69.44% disease incidence of *Fusarium* wilt under pot culture studies. Singh et al. (2011) carried out an experiment for pathogenecity in different isolates of *F. oxysporum* f.sp. *lycopersici* and the result showed that 88.8 per cent in virulent isolates. Artificial inoculation of wilt pathogen expressed the symptom significantly and the incidence of 78.50% in tomato plants was observed by Shanmugam and Kanoujia (2011). There was variation in the incidence of wilt between various isolates which was reported by Abdel-Monaim (2012) indicated that isolates FT1 recorded 43.4% and FT7 showed 46.5% wilt under glass house conditions. Sundaramoorthy and Balabaskar (2013) artificially inoculated the *Fusarium* wilt pathogen in tomato plants under controlled environment and they observed incidence of wilt disease up to 57.73%.

The amplification of IGS region using PCR has found broadspectrum application in fungal diagnosis and detection (Louis et al., 2000). These IGS regions are very useful to identify the fungal occurrence, especially when coupled with preliminary symptom observation on the field (Kawabe et al., 2005). In the present study, IGS primers were used to identify the *F. oxysporum* f.sp *lycopersici*. This study was supported by Balogun et al. (2008), who reported that, IGS region helps to detect the fungal in the host, especially when the symptoms are not yet manifest.

Studying protein profiles of pathogens would provide information about the variation in protein expression levels in between the isolates. It would also help in the identification of common proteins within the population of particular pathogens. Several workers have studied the morphology of the fungus and genetic diversity between the *F*. *oxysporum* f.sp. *lycopersici* isolates (Mishra et al., 2010; Taheri et al., 2010; Nirmaladevi et al., 2016). However, not much work has been reported on the protein profiles of this pathogen. In the present study, the protein profiles of different isolates of *Fusarium* wilt pathogen were analyzed by SDS-PAGE. Results showed that although certain isolates were morphologicaly similar in culture, the proteins expressed on SDS gels were different. This variation in the protein profile suggests a possible reason for the variation in level of pathogenicity between the isolates.

Similar results were reported by Aly et al. (2003), including profiles of protein data that can clearly differentiate isolates of *Fusarium* spp. with a few exceptions. In the other hand, Belisario et al. (1998) compared the protein profiles from total mycelium and found differences in species and formae speciales *viz., F. oxysporum, F. solani* and *F. culmorum* using SDS-PAGE analysis. Hassan et al. (2008) used SDS-PAGE analysis to differentiate among the isolates of *Fusarium* spp.

Now a day's fungal proteomics become an appropriate tool for obtaining molecular map about pathogenicity and factors involved in virulence, thus opening up new insights for plant pathogen detection and protection. It is assumed that proteins are controlling factor for pathogenicity of fungi. Fungus of phytopathogens secretes a broad spectrum of proteins which are playing a crucial role their virulence, pathogenicity and enable their parasitic lifestyle. Among them, most important one is cell wall-degrading enzymes and effectors and they accelerate biochemical, physiological, and morphological responses in host plants to facilitate infection (Bhadauria et al., 2010). In this regards, identification of the virulence responsive proteins is most important one for uncovering the mechanism of pathogenic process in plants.

In the present study, proteomics approach was used to identify differentially expressed proteins in virulent isolate (FOL-8) and less virulent (FOL-20) isolates responsible for disease development and their virulence. From the identified proteins, two proteins namely F8 and F9 with the molecular weight of 22 and 28 kDa were found to be significantly down regulated and proteins with pI range from 4.8 to 5.4 were found to be upregulated in virulent isolates (FOL-8). This type of findings of was supported by several workers. Young et al. (2001) extracted the proteins from *F*. *oxysporum* f.sp. *cubense* and identified differentially expressed proteins which were involved in host infections. Proteome analysis in *F. oxysporum* showed the role of Fb1 protein in proteasome degradation and susceptible as potential targets of ubiquitination (Miguel-Rojas and Hera, 2013). Similarly, Sun et al. (2014) studied the proteomic changes in *F. oxysporum* race 1 and race 4 through 2-D PAGE. They found 37 proteins which were differentially expressed between the races and they were involved in various biological functions including the pathogenicity. Singh et al. (2017) studied the protein profiling of *Fusarium* wilt pathogen and confirmed that the differentially expressed proteins were ultimately involved in the cellular physiology for disease development.

In this research, 10 differentially expressed proteins were analyzed and identified. The function of proteins has been reported by several workers as follows. In *Aspergillus niger*, a genome-wide transcriptional analysis showed that during stress conditions genes encodes chaperone proteins LHS1 and KAR2 which were significantly up regulated. These chaperone proteins essential for asexual development of *Magnaporthe oryzae* and its infection in rice (Guillemette et al., 2007; Yi et al., 2009). Flavin adenine dinucleotide (FAD) is one of the important cofactor involved in electron transfer of flavoprotein thus provides abundant energy during mycelia formation and fruiting bodies as well as in other biological insights. Meng et al. (2013) suggested that differential expression of FAD-binding proteins may played crucial role in *Volvariella volvacea* for revealing the distinction between homokaryons and heterokaryons.

Six FAD binding domain containing proteins in *Moniliophthora roreri* (frosty pod rot disease) were expressed during the necrotrophic phase which is associated with fungal growth/pathogenicity and nutrient acquisition (Meinhardt et al., 2014). A number of proteins involved in the transport of sulfate across the membranes (Sebastian et al., 2007). Hence our results suggest that, up regulation of sulfate anion transporter may help higher uptakes of sulfate ions by virulent isolate for their growth and development.

The fungal protein, cytochrome P450 monooxygenases are very essential factor for cellular processes and play different roles. It catalyze the conversion of hydrophobic intermediates in both primary and secondary metabolic pathways followed by allowing of fungi to grow under different environmental conditions. Cresnal and Petric (2011) reported that cytochrome P450 enzymes are essential for primary metabolisms, preserve cell wall integrity and to form the spore outer wall regardless of the fungus morphology. From this information our results suggest that presence of higher level expression of cytochrome P450 enhanced the cell wall integrity and morphology of virulent isolate.

Production of extracellular degradative enzymes by the plant pathogenic fungus, play an important role in pathogenesis. They also penetrate the cuticle by the enzymes cutinase, which hydrolyses cutin, facilitating fungus for penetration. The cutin monomers production from the cuticle by little amounts of cutinase on fungal spore surfaces can greatly increase the amount of cutinase secreted by the spore. Many reports are supports the crucial role of cutinase in host penetration by number of fungi (Kolattukudy et al., 1995). Skamnioti and Gurr (2007) reported that cutinase required for appressorium formation in *Magnaporthe grisea* which mediates host penetration and their virulence. Likewise, Secretion of fusaric acid at the subcellular level, affects various biochemical processes correlated to membrane permeability changes, mitochondrial dysfunctions, and arrests the rate of respiration and resulted in death of cell (Singh et al., 2017). From this information, it revealed that expression of cutinase in virulent isolate involved in penetration and development of pathogenicity in tomato plants.

In the other hand, two proteins namely F8 and F9 were down regulated in virulent isolate. Functions of this protein revealed that they were involved in detoxification, hydrolysis or secondary metabolites biosynthesis, involved in transcription elongation and maintenance of genome integrity. These types of results were supported by several workers. Gao et al. (2014) reported that, glycoside hydrolases very essential for detoxification, hydrolysis or secondary metabolites biosynthesis and play a crucial for *Curvularia lunata* survival in varied stress environments. Similar type of protein was observed in *M. anisopilae* required to cause disease in insect (Zheng et al., 2011).

Over all view of our fungal proteomics study revealed that up regulation of proteins clearly involved in more pathogenicity as well as virulence. Presence of these proteins in virulent isolate (FOL-8) was responsible for maintain the population, morphology, fast growth, plant cell wall degradation, and over comes the host defense mechanism. Hence the present study provided information about the mechanism of virulence behind disease severity in tomato plants.

## Author contributions

RM: Performed the laboratory experiments, statistical analysis, data recording, and manuscript write-up; SH and GK: co-supervised the study; TR: Outlined the experiment, inspected the entire study and created two dimensional electrophoresis facilities for the study. All authors proofread and reviewed the manuscript.

### Conflict of interest statement

The authors declare that the research was conducted in the absence of any commercial or financial relationships that could be construed as a potential conflict of interest.
